# Analyzing the impact of 23 mg/day donepezil on language dysfunction in moderate to severe Alzheimer's disease

**DOI:** 10.1186/alzrt84

**Published:** 2011-06-20

**Authors:** Steven H Ferris, Frederick A Schmitt, Judith Saxton, Sharon Richardson, Joan Mackell, Yijun Sun, Yikang Xu

**Affiliations:** 1 Alzheimer Disease Center, Centre of Excellence on Brain Aging, New York University Langone Medical Center, 145 East 32nd Street, New York, NY 10016, USA; 2 Sanders-Brown Center on Aging/Alzheimer's Disease Center, University of Kentucky, 800 South Limestone Street, Lexington, KY 40536-0230, USA; 3 Alzheimer's Disease Research Center, University of Pittsburgh Medical Center, 3459 Fifth Avenue, Pittsburgh, PA 15213, USA; 4 Medical Affairs, Eisai Inc., 100 Tice Boulevard, Woodcliff Lake, NJ 07677, USA; 5 Medical Affairs, Pfizer Inc, 235 East 42nd Street, New York, NY 10017, USA; 6 Biometrics Department, Eisai Inc., 100 Tice Boulevard, Woodcliff Lake, NJ 07677, USA; 7 Biometrics Department, Pfizer Inc, 235 East 42nd Street, New York, NY 10017, USA

## Abstract

**Introduction:**

Progressive language impairment is among the primary components of cognitive decline in Alzheimer's disease (AD). Because expressive and receptive language help to maintain emotional connections to caregivers and support the management of AD patients' functional needs, language plays a critical role in patients' emotional and physical health. Using data from a large prospective clinical trial comparing two doses of donepezil in patients with moderate to severe AD, we performed a *post hoc *analysis to determine whether a higher dose of donepezil was associated with greater benefits in language function.

**Methods:**

In the original randomized, double-blind clinical trial, 1,467 patients with moderate to severe AD (baseline Mini-Mental State Examination (MMSE) score 0 to 20) were randomized 2:1 to receive donepezil 23 mg/day or to continue on donepezil 10 mg/day for 24 weeks. In this *post hoc *analysis, the Severe Impairment Battery-Language scale (SIB-L) and a new 21-item SIB-derived language scale (SIB[lang]) were used to explore differences in language function between the treatment groups. Correlations between SIB-L and SIB[lang] scores and scores on the severe version of the Alzheimer's Disease Cooperative Study-Activities of Daily Living inventory (ADCS-ADL-sev), the Clinician's Interview-Based Impression of Severity-plus caregiver input/Clinician's Interview-Based Impression of Change-plus caregiver input (CIBIS-plus/CIBIC-plus) and the MMSE were also investigated.

**Results:**

At week 24, treatment with donepezil 23 mg/day was associated with an improvement in language in the full intention-to-treat population, whereas language function declined in the group treated with donepezil 10 mg/day (SIB-L treatment difference 0.8, *P *= 0.0013; SIB[lang] treatment difference 0.8, *P *= 0.0009). Similar results were observed in a cohort of patients with more severe baseline disease (MMSE score 0 to 16). At baseline and week 24, correlations between the SIB-derived language scales and the ADCS-ADL-sev and CIBIC-plus were moderate, but the correlations were stronger between the language scales and the MMSE scores.

**Conclusions:**

Patients with moderate to severe AD receiving donepezil 23 mg/day showed greater language benefits than those receiving donepezil 10 mg/day as measured by SIB-derived language assessments. Increasing the dose of donepezil to 23 mg/day may provide language benefits in patients with moderate to severe AD, for whom preservation of language abilities is especially critical.

ClinicalTrials.gov identifier: NCT00478205

## Introduction

An estimated 5.1 million Americans over 65 years of age have Alzheimer's disease (AD), and more than half of these individuals are classified as having moderate or severe disease [1]. As disease severity increases, patients progressively lose the ability to communicate, express their needs and participate in their accustomed relationships [2-4]. In patients with more advanced disease, this often leads to an inability to sustain social relationships, express needs for medical attention and relate or respond to caregivers. Moreover, caregivers often become frustrated by difficulties in understanding and meeting patients' loved ones' needs, which can lead to further patient distress [3,5-7]. The ability to use language to communicate helps to maintain emotional connections between the patient and his or her caregiver and/or family, and loss of language ability is among the most distressing factors associated with AD [3]. Thus, treatment that can delay or even improve language abilities is an important focus of AD therapy.

Effective treatment of the loss of language abilities presupposes the availability of reliable instruments for evaluating such function. Commonly used tools such as the Alzheimer's Disease Assessment Scale-cognitive subscale, which is primarily a clinical trial measure developed to track the progression of AD symptoms [8], and the Mini-Mental State Examination (MMSE) [9], which is used in both clinical trials and clinical practice, are valid and reliable measures of cognition and change in cognitive function in less advanced stages of AD. However, when patients progress into the moderate to severe stages of AD, these common tools are subject to a "floor effect," as they may become relatively insensitive to changes in disease severity [10]. The Severe Impairment Battery (SIB), originally developed more than 20 years ago, was designed to assess cognition and changes in cognitive function in patients with more advanced impairment [11-14]. The SIB uses one-step conversational commands presented in conjunction with gestural cues, enhancing its utility in patients with profound impairments in communicative ability. The SIB remains a reliable and sensitive assessment tool as patients progress along the continuum of moderate to severe AD, and it is a widely used and validated measure of cognitive function in clinical trials [10,15-17].

Because of the centrality of language to cognitive function, 24 of the 51 total items and subitems in the full SIB scale assess language ability and comprise the SIB language subscale [3]. In a previous study, a principal components factor analysis of these 24 items was performed to determine which were the most relevant in assessing language function [3]. Using baseline data from patients enrolled in four placebo-controlled trials of memantine, an *N*-methyl-D-aspartate receptor antagonist, in moderate to severe AD (baseline MMSE score <15), 21 of the 24 items were found to have a factor loading >0.5 and were subsequently included in a new language scale, the SIB Language scale (SIB-L). Three of the twenty-four items had a factor loading <0.5 and were excluded from the SIB-L; the maximal SIB-L score is 41. A subsequent *post hoc *analysis of data from the same four trials, but which excluded patients who did not have language deficits at baseline, demonstrated significant differences on the SIB-L favoring active treatment over placebo, but SIB-L scores were decreased compared with baseline in both treatment arms [18].

The cholinesterase inhibitor donepezil is the most frequently prescribed medication worldwide for the symptomatic treatment of mild, moderate and severe AD [19,20]. Recently, a new dose of donepezil, a 23-mg tablet, was approved by the US Food and Drug Administration for the treatment of moderate to severe AD. Herein we report the results of a *post hoc *analysis of data from a large, multinational, double-blind trial of donepezil 23 mg/day versus donepezil 10 mg/day performed to determine whether treatment with higher-dose donepezil is associated with benefits in language abilities in patients with moderate to severe AD. Moreover, since previous reports have indicated that cognition and function may be interrelated in patients with moderate to severe AD [21,22], we also evaluated relationships between measures of language function and other instruments used to assess AD status.

## Materials and methods

### Study design

Detailed methods used in the original clinical trial have been described previously [23]. In brief, this was a 24-week, double-blind, parallel group trial including 1,467 patients with moderate to severe AD (baseline MMSE scores 0 to 20 inclusive) who had been receiving a stable dose of donepezil 10 mg/day for at least three months. Patients were randomized in a 2:1 ratio either to an increased donepezil dose of 23 mg/day or to continue their preexisting donepezil 10 mg/day dose. The duration of treatment was 24 weeks. Coprimary efficacy measures were change from baseline in SIB total score (cognition) and the Clinician's Interview-Based Impression of Change-plus caregiver input (CIBIC-plus; global function) score at week 24. The protocol and informed consent form for the original trial were approved by the independent ethics committee and institutional review board of each participating site and conformed to the principles of the Declaration of Helsinki and all local regulations. Written informed consent was obtained from each participating patient, if possible, or from the patient's legal guardian or representative. If a patient was unable to provide written consent, verbal assent to participate was required, with documentation of this assent noted in the study record [23].

### Scale construction

A new SIB-derived language scale, the 21-item Severe Impairment Battery-derived language scale (SIB[lang] scale), was constructed by performing a principal components factor analysis, similar to that employed in the creation of the SIB-L [3], on baseline scores of the 24-item SIB language subscale in the full intention-to-treat (ITT) population (see Additional File 1 for a listing of the 24 items in the SIB language subscale). Initial factors were selected on the basis of eigenvalues >1. Selection of items for inclusion in the SIB[lang] scale was based on a threshold of 0.5 for the single-item loading of each identified factor. Items with a factor loading <0.5 were deleted. In total, 21 of the 24 items in the SIB language subscale were identified as having a factor loading >0.5 and were included in the SIB[lang] scale. Three items were excluded because of a factor loading <0.5: free discourse, verbal comprehension and repeating the word "baby." As with the SIB-L scale, scores on the SIB[lang] scale range from 0 to 41.

### *Post hoc *analysis population

The *post hoc *language analyses were performed in the full ITT population (all MMSE scores 0 to 20) and in a cohort of patients with more severe baseline disease (MMSE scores 0 to 16).

### Analysis of treatment effects

Changes from baseline to week 24 in SIB-L and SIB[lang] scale scores for the 23 mg/day and 10 mg/day treatment groups were analyzed using an analysis of covariance model with terms for baseline score, country and treatment. For each end point, the least squares (LS) or adjusted mean for each treatment group was calculated, as were between-treatment differences (donepezil 23 mg/day compared with donepezil 10 mg/day) in adjusted means, 95% confidence intervals (95% CIs) for the differences and the *P *values for the between-treatment differences. Summary statistics (median, mean, standard deviation (SD), minimum, maximum and number of patients with nonmissing data) at week 24 using last observation carried forward and observed cases are provided by treatment group.

### Analysis of correlations between outcome measures

Relationships between SIB-L and SIB[lang] scale scores and scores on the MMSE, the Clinician's Interview-Based Impression of Severity-plus caregiver input (CIBIS-plus; the baseline version of the CIBIC-plus assessment tool) and CIBIC-plus, and the severe version of the Alzheimer's Disease Cooperative Study-Activities of Daily Living (ADCS-ADL-sev) were examined. The ADCS-ADL-sev scale was analyzed both as a complete unit and when separated into two subscales reflecting basic and instrumental ADL abilities. Pearson correlation coefficients were calculated for baseline, week 24 and change from baseline scores. Because higher scores on the SIB-derived language scales, the MMSE and the ADCS-ADL-sev indicate less impairment, correlation coefficients between these measures are positive. Since higher scores on the CIBIS-plus/CIBIC-plus scales indicate greater impairment, correlation coefficients for relationships between SIB-L or SIB[lang] scores and CIBIS-plus/CIBIC-plus scores are negative.

## Results

### Patients

The full ITT population comprised 1,371 patients, 909 of whom received donepezil 23 mg/day and 462 of whom received donepezil 10 mg/day. Demographics and baseline disease characteristics were similar for both donepezil treatment groups (Table 1), and no meaningful differences were observed between the groups in relation to baseline scores on the SIB-derived language scales (Table 2).

**Table 1:** Demographics and baseline characteristics in the intention-to-treat population^a^

Characteristics	Donepezil 23 mg/day	Donepezil 10 mg/day
Age, years		
Number of patients	909	462
Mean (±SD)	73.8 (8.48)	73.8 (8.55)
Gender		
Number of patients	909	462
Males, *n *(%)	335 (36.9%)	175 (37.9%)
Females, *n *(%)	574 (63.1%)	287 (62.1%)
MMSE		
Number of patients	908	462
Mean (±SD)	13.1 (4.99)	13.1 (4.72)
ADCS-ADL-sev total		
Number of patients	908	461
Mean (±SD)	34.1 (10.88)	34.5 (11.19)
SIB		
Number of patients	907	462
Mean (±SD)	74.2 (17.58)	75.6 (16.28)
CIBIS-plus		
Number of patients	904	461
Mean (±SD)	4.42 (0.85)	4.38 (0.89)
Concomitant memantine use		
Number of patients	909	462
Using memantine, *n *(%)	338 (37.2)	163 (35.3)

^a^MMSE = Mini-Mental State Examination; ADCS-ADL-sev = Alzheimer's Disease Cooperative Study-Activities of Daily Living inventory, severe version; SIB = Severe Impairment Battery; CIBIS-plus = Clinician's Interview-Based Impression of Severity-plus caregiver input; SD = standard deviation.

**Table 2:** Baseline scores on SIB-derived language scales in the ITT population^a^

MMSE score	SIB-L		SIB[lang]	
	**Donepezil 23 mg/day**	**Donepezil 10 mg/day**	**Donepezil 23 mg/day**	**Donepezil 10 mg/day**

0 to 20^b^	32.4 ± 8.2 (*n *= 907)	32.9 ± 7.7 (*n *= 462)	31.4 ± 8.3 (*n *= 907)	31.9 ± 7.7 (*n *= 462)
0 to 16	30.5 ± 8.9 (*n *= 641)	31.2 ± 8.3 (*n *= 331 )	29.5 ± 9.0 (*n *= 641)	30.3 ± 8.4 (*n *= 331)

^a^SIB = Severe Impairment Battery; ITT = intention to treat; MMSE = Mini-Mental State Examination; SIB-L = Severe Impairment Battery-Language scale; SIB[lang] = 21-item Severe Impairment Battery-derived language scale; ^b^full ITT population. Data are means ± SD.

### Study coprimary end points

As previously reported, the change from baseline in SIB scores at week 24 showed significant improvement in cognition associated with treatment with donepezil 23 mg/day relative to treatment with donepezil 10 mg/day (LS mean score change 2.6 versus 0.4, treatment difference 2.2; *P *< 0.001) [23]. Treatment difference in global function as measured by the CIBIC-plus scale numerically favored donepezil 23 mg/day but was not statistically significant.

### *Post hoc *analysis: treatment effects

**Figure 1:**
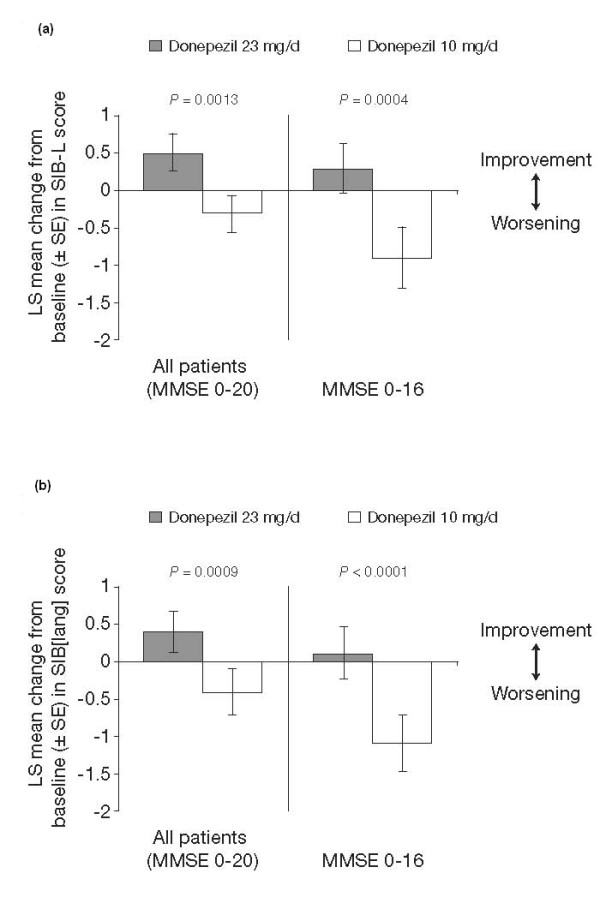
**Least squares (LS) mean change from baseline to week 24 in Severe Impairment Battery-Language scale (SIB-L) scores (A) and 21-item Severe Impairment Battery-derived language scale (SIB[lang]) scores (B)**. MMSE = Mini-Mental State Examination; SE = standard error of the mean.

Changes in SIB-L and SIB[lang] scores from baseline to week 24 are shown in Figure 1. For both language assessments, donepezil 23 mg/day was associated with improvement in language function in the full ITT population as well as in the MMSE scale 0 to 16 score cohort. Regardless of the language assessment used, treatment with donepezil 10 mg/day was associated with a mean decline in language function in both the full ITT and MMSE scale 0 to 16 score cohort. Treatment differences between donepezil 23 mg/day and donepezil 10 mg/day were statistically significant in all analyses (Figure 1).

### *Post hoc *analysis: correlations

At baseline, SIB-L and SIB[lang] scores were moderately correlated with scores on the ADCS-ADL-sev (*r *≈ 0.5) and CIBIS-plus (*r *≈ -0.5) (Table 3). Baseline correlations between SIB-L and SIB[lang] scores and scores on the MMSE were strong (*r *≈ 0.7).

**Table 3:** Correlations between SIB-L and SIB[lang] scores and scores on the ADCS-ADL-sev (total, basic and instrumental), CIBIS/CIBIC-plus and MMSE (full ITT population)^a^

	SIB-L			SIB[lang]		
AD scales	Baseline	Week 24	Change from baseline	Baseline	Week 24	Change from baseline
ADCS-ADL-sev total						
Baseline	0.5268		0.1497	0.5250		0.1418
Week 24		0.5820			0.5777	
Change from baseline			0.2837			0.2912
ADCS-ADL-sev basic						
Baseline	0.5214		0.1124	0.5193		0.1036
Week 24		0.5669			0.5621	
Change from baseline			0.2643			0.2695
ADCS-ADL-sev instrumental						
Baseline	0.4745		0.1546	0.4730		0.1481
Week 24		0.5400			0.5365	
Change from baseline			0.2369			0.2441
CIBIS-plus/CIBIC-plus						
Baseline (CIBIS-plus)	-0.5100		-0.1722	-0.5117		-0.1821
Week 24 (CIBIC-plus)		-0.1918			-0.1891	
Change from baseline (CIBIC-plus)			-0.3484			-0.3493
MMSE						
Baseline	0.7208		0.2205	0.7183		0.2359
Week 24		0.7375			0.7394	
Change from baseline			0.3618			0.3517

^a^SIB-L = Severe Impairment Battery-Language scale; SIB[lang] = 21-item Severe Impairment Battery-derived language scale; ADCS-ADL-sev = Alzheimer's Disease Cooperative Study-Activities of Daily Living inventory, severe version; CIBIS-plus = Clinician's Interview-Based Impression of Severity-plus caregiver input; CIBIC-plus = Clinician's Interview-Based Impression of Change-plus caregiver input; MMSE = Mini-Mental State Examination; ITT = intention to treat; *n *values for individual correlation coefficients ranged from 1,363 to 1,371. All correlations were statistically significant (*P *≤ 0.0001).

At week 24, correlations between SIB-L and SIB[lang] scores and scores on the ADCS-ADL-sev and MMSE were marginally but consistently stronger than at baseline (Table 3). In contrast, correlations between scores on the SIB-derived language scales and CIBIS-plus/CIBIC-plus scores were weaker at week 24 than at baseline.

Correlations between change from baseline in SIB-L and SIB[lang] scores and change from baseline in scores on other assessments were generally of weak to moderate strength, with the MMSE scores showing the strongest correlation (Table 3). Likewise, change from baseline in SIB-L and SIB[lang] scores showed only weak correlations with baseline assessments for the other measures.

## Discussion

The already fragile relationships in the lives of patients with AD can become profoundly disrupted as patients lose the ability to communicate coherently. Attachment to caregivers and others becomes compromised, and the effect of such detachment on caregivers can be deleterious. Furthermore, it is difficult for clinicians and caregivers to render optimal care in the absence of clear input from patients regarding what they are feeling or concerned about. When language comprehension deteriorates, it is also likely to further increase caregiving stress, as patients have difficulty understanding conversation and even simple directions. Moreover, decline in language is associated with increased risk of mortality [24]. Therefore, therapy to slow or reduce the loss of language abilities has the potential to result in additional quality time for patients and caregivers alike.

In the primary study of donepezil 23 mg/day versus donepezil 10 mg/day, patients treated with donepezil 23 mg/day showed significant improvement in cognition, as measured by the SIB, compared with patients taking donepezil 10 mg/day [23]. Notably, SIB total scores at week 24 were improved relative to baseline scores in the donepezil 23 mg/day group, indicating that there was no measurable decline in cognition over the six months of treatment. In this *post hoc *analysis, we found that treatment with donepezil 23 mg/day resulted in significant improvement in language ability compared with donepezil 10 mg/day in patients with moderate to severe AD as measured by the two SIB-derived language scales: the SIB-L, and the SIB[lang].

Since the SIB-L was derived using data from a population of patients with baseline MMSE scores <15 enrolled in studies of memantine versus placebo [3], there was a possibility that this scale would not be suitable for the measurement of language function in the current analysis, as this patient population had differing baseline characteristics and demographics. Therefore, the SIB[lang] scale was created by performing a factor analysis of baseline SIB language subscale scores in the full ITT population from the study of donepezil 23 mg/day versus donepezil 10 mg/day. As was the case in the creation of the SIB-L [3], the SIB[lang] scale comprised 21 of the 24 items in the SIB language subscale, with three items excluded. However, while the free discourse item was excluded from both scales, the other two items eliminated in the creation of the SIB[lang] scale (verbal comprehension and repeating the word "baby") were different from those excluded from the original SIB-L (forced choice naming, cup and shape identification, square). This suggests that the results of the two factor analyses were somewhat study population-driven, likely reflecting AD severity as well as cultural influences on language assessment. Nevertheless, the fact that the SIB-L and SIB[lang] scales showed equivalent performance in the current analysis demonstrates that the utility of these language scales extends beyond the specific patient populations from which they are derived.

The findings of this *post hoc *language analysis have important clinical implications. One is to support the assertion that language and the ability to communicate can be reliably and easily assessed in patients with moderate to severe AD using SIB-derived language scales. Of potentially greater importance is the finding that not only were substantial incremental benefits in language ability achieved with donepezil 23 mg/day treatment beyond those achieved with donepezil 10 mg/day, but also the benefits were evident in both the full study population (MMSE scores 0 to 20) and the cohort of patients with more severe baseline disease (MMSE scores 0 to 16). Indeed, in both analysis populations, the donepezil 23 mg/day treatment group showed improvements in language function above baseline performance levels after six months of treatment, whereas the donepezil 10 mg/day group showed declines in language function. This suggests that the donepezil 23 mg/day dose may help to preserve language abilities in patients as they move across the spectrum of moderate to severe AD.

Why the donepezil 23 mg/day dose should have a greater effect on language function as compared with the donepezil 10 mg/day dose was not investigated in this analysis. However, one could speculate that the observed outcomes may be due to the higher dose of donepezil providing greater enhancement of cholinergic function in regions of the brain controlling speech and language. Another hypothetical scenario is that the observed language benefits are driven in whole or in part by enhanced effects of the 23 mg/day dose on regions of the brain controlling other cognitive processes, such as attention and memory. Indeed, it is logical that attention deficits could substantially influence a patient's language ability as measured by the SIB-L and SIB[lang] scales. Moreover, there is clearly some overlap between certain aspects of language impairment, such as word-finding difficulty, and more general memory retrieval problems. Nevertheless, the items included in the SIB-L and SIB[lang] scales cover a broad range of language functions, including processes that are not highly dependent on memory, and, as such, treatment effects on memory are likely to account for a relatively small portion of the observed language improvements. Ultimately, the direct and indirect mechanisms whereby donepezil 23 mg/day provides improved language benefits over the donepezil 10 mg/day dose need to be determined in adequately designed prospective studies.

Language effects of other pharmacologic treatments for AD have varied in studies of patients with moderate to severe disease. For example, in an analysis of pooled data from four memantine trials in patients with moderate to severe AD (MMSE score <15), mean SIB-L score changes from baseline through week 24 or week 28 were significantly better for those receiving the active treatment compared with placebo, but mean SIB-L scores declined in both treatment groups [18]. Likewise, in a *post hoc *analysis of the specific language effects of memantine in patients with moderate to severe AD (MMSE scores 3 to 14) enrolled in six clinical trials, memantine was associated with significantly better SIB language subscale scores compared with placebo; however, scores in both groups declined from baseline to the end of the study (six months) [25]. In addition, in a cohort of patients with severe AD (MMSE scores 5 to 12) treated with the cholinesterase inhibitor galantamine for 26 weeks, mean SIB language subscale scores were numerically improved at the end point, but improvement over placebo was not statistically significant [26].

Not surprisingly, the MMSE, a measure of cognition that includes several language items, showed the strongest correlation with SIB-L and SIB[lang] scores for all relationships explored. However, correlations between the language scales and the MMSE were substantially stronger for baseline and week 24 scores (*r *≈ 0.7) than for change from baseline scores (*r *≈ 0.35). This suggests that cognition as measured by the MMSE is strongly related to language abilities as measured by SIB-derived language scales, but that changes in cognition on the MMSE and changes in language on the SIB language scales track differently over time. Pearson correlation coefficients also showed that baseline SIB-L and SIB[lang] scores were moderately correlated with baseline scores for the ADCS-ADL-sev and CIBIS-plus, suggesting that language abilities and functional abilities may be interrelated in the moderate to severe AD population. However, correlations for change from baseline scores were only of moderate to weak strength. As Farlow and colleagues [23] previously reported, changes in ADCS-ADL-sev scores and CIBIC-plus scores in this study were small. Coupled with the limitations of these classes of instruments, this may at least in part explain the absence of stronger correlations. It is also noteworthy that changes from baseline to week 24 in SIB-L and SIB[lang] scores were only weakly correlated with baseline scores on the ADCS-ADL-sev, CIBIS-plus and MMSE. This suggests that there is little relationship between baseline functional or cognitive status and treatment-mediated changes in language abilities; thus, patients with AD may achieve meaningful language benefits with treatment, irrespective of the degree of functional or cognitive impairment. Since the correlation findings are from a *post hoc *analysis, they will need to be corroborated in prospective studies.

## Conclusions

The results of this analysis suggest that donepezil 23 mg/day may result in improvements in the critical factor of language in advanced AD as measured by SIB-derived language scales. These findings indicate that increasing the dose of donepezil to 23 mg/day may provide language benefits in patients with moderate to severe AD, for whom preservation of language abilities is especially critical. Supporting communication in patients with a progressive disease such as AD may have the potential, even in advanced stages of AD, to improve patients' quality of life and reduce the burden of AD experienced by caregivers.

## Abbreviations

AD: Alzheimer's disease; ADCS-ADL-sev: Alzheimer's Disease Cooperative Study-Activities of Daily Living inventory; CIBIC-plus: Clinician's Interview-Based Impression of Change-plus caregiver input; CIBIS-plus: Clinician's Interview-Based Impression of Severity-plus caregiver input; ITT: intention to treat; LOCF: last observation carried forward; LS: least squares; MMSE: Mini-Mental State Examination; SD: standard deviation; SIB: Severe Impairment Battery; SIB-L: Severe Impairment Battery-Language scale; SIB[lang]: 21-item Severe Impairment Battery-derived language scale.

## Competing interests

The analyses described in this article derive from a phase III clinical study (ClinicalTrials.gov identifier: NCT00478205) that was sponsored by Eisai Inc. Editorial assistance was provided by PAREXEL Inc. and was funded by Eisai Inc. and Pfizer Inc. The article-processing charge was financed by Eisai Inc. and Pfizer Inc. SHF has served as a paid scientific consultant to Pfizer Inc related to donepezil and several investigational compounds (≤$10,000/year; there was no payment for participation in this article). His institution has received grant/contract support from Pfizer Inc for the conduct of clinical trials of dementia and to support educational programs. He has also served as a scientific consultant to other companies marketing or developing drugs for cognition, including Lundbeck, Elan, Janssen AI, Eli Lilly, Merz, Novartis, Toyama, Bellus Health, Bayer Healthcare, Accera, Otsuka, Sunovion, Symphony Icon and Torrey Pines, and his institution has received grant/contract support for clinical trials from Pfizer, Eli Lilly, Janssen AI, Baxter and Bristol-Myers Squibb. He also has stock options from Accera, Intellect Neurosciences, MedAvante and Symphony Icon. FAS has no financial competing interests to report. He has served on a Data Safety Monitoring Committee for Pfizer Inc for which the University of Kentucky received reimbursement (<$4,000). JS has been paid as a consultant to Eisai Inc., Pfizer Inc, Forest Pharmaceuticals and Novartis. JS currently receives royalties from the original Severe Impairment Battery on which this reduced scale is based. SR is an ex-employee of Eisai Inc. JM is an employee of Pfizer Inc. YS is an employee of Eisai Inc. YX was an employee of Pfizer Inc.

## Authors' contributions

SHF, FAS, SR and JM helped to conceive and design the described analyses, participated in the data analysis and assisted in the drafting, editing and interpretation of the manuscript. JS developed the original Severe Impairment Battery scale, helped to conceive and design the described analyses, participated in the data analysis and assisted in the drafting, editing and interpretation of the manuscript. YS and YX assisted in the statistical planning and performance of the *post hoc *analyses and assisted in the drafting, editing and interpretation of the manuscript. All authors read and approved the final version of the manuscript.

## Supplementary Material

Additional file 1 **Items in the SIB Language Subscale**. A table listing the 24 items in the Severe Impairment Battery (SIB) language subscale. Click here for file
