# Supplementary material for: Analyzing the impact of 23 mg/day donepezil on language dysfunction in moderate to severe Alzheimer's disease
Source: Alzheimers Res Ther. 2011 Jun 20;3(3):22. doi: 10.1186/alzrt84 (PMC3226311; doi:10.1186/alzrt84)
Supplement: Additional file 1 — Items in the SIB Language Subscale. A table listing the 24 items in the Severe Impairment Battery (SIB) language subscale. [file alzrt84-S1.PDF]

Additional File 1 - Items in the SIB Language Subscale

**Item**	**Category**
4a: Write name	Writing
4b: Copy name	
9a: Reading comprehension	Reading
9c: Reading	
9b: Verbal comprehension	Comprehension
13: Fluency	Verbal fluency
6: Months of year	Naming
8a: Responsive naming—cup	
8b: Responsive naming—spoon	
30a: Color naming—red	
30b: Color naming—green	
34a: Shape identification—circle	
34b: Shape identification—triangle	
20: Confrontation naming—spoon	
22: Object naming—spoon	
24: Forced choice naming—spoon	
15: Confrontation naming—cup	
17: Object naming—cup	
26: Color naming—blue	
19: Forced choice naming—cup	
30c: Shape identification—square	
11a: Repeating “people spend money”	Repetition
11b: Repeating “baby”	
40: Free discourse	Discourse
